# Abstract of Proceedings of the Buffalo Medical Association

**Published:** 1868-04

**Authors:** T. M. Johnson

**Affiliations:** Sec’y.


BUFFALO

### Medical and Surgical Journal.

VOL. VII. APRIL, 1868. No. 9.

### Original Communications.

ART. I.—*Abstract of Proceedings of the Buffalo Medical Association.*

TUESDAY EVENING, February 4th, 1868.

On motion of Dr. Johnson, Dr. J. F. Miner was elected Chairman of the meeting. Members present—Drs. Miner, Little, C. F. A. Nichell, Mackay, Abbott, Edmonds, Gay, Kamerling, Henry Nichell, Eastman, Wetmore and Johnson.

The minutes of the last meeting were read and approved.

Dr. Henry Nichell read the following Essay upon the treatment of Asiatic Cholera:

*Mr. President and Gentlemen of the Buffalo Medical Association:*

In a former meeting you have appointed me to the honor of reading an Essay on some medical theme this evening. In compliance with your invitation I shall propose to you, observations and facts concerning a certain plan of treatment of Asiatic cholera adopted by the writer during the epidemics of 1852 and 1854, in the city of Buffalo. I sincerely hope the subject selected, although it may perhaps dissappoint your just expectations, is yet worthy of your earnest and thoughtful consideration.

All modes devised for the prevention and cure of this formidable malady, have been censured by modern and quite recent writers, more or less severely, and I may add in part, perhaps, justly. It was the late venerable Dr. Velpeau, who boldly declared before the Academy of Medicine in Paris, "that we know nothing more of treatment of cholera now than on its first appearance in 1832." "All our remedies and modes of practice," he says, "have failed." You will, therefore, pardon me when I frankly confess that it is not my fortune to offer to you any superior method or plan as regards the treatment of the disease in question, but to present to you simply "observations and facts" about what had been termed the treatment of cholera with calomel and opium, as employed in the second stage, and also to a certain extent, in the state of collapse of epidemic cholera. This mode of treatment has of late been bitterly attacked and condemned by some eminent authorities, although not a few articles upon the subject also show its enthusiastic advocates and able defenders.

Dr. R. Nelson, in his monograph on Asiatic cholera, page 174, does not hesitate to use the following language about physicians prescribing such agents, especially calomel:

"So potent were a wrong education, a defective physiology, a badly acquired habit, a blind faith in false doctrines, and a pernicious obstinacy in adhering to habit, that almost all physicians made use of calomel. One gave a grain, with or without opium, every half an hour, another two or five grains, finding these doses inefficient, ten to twenty grains; others, bolder, gave half a drachm, one or two drachm doses. Should one or two patients out of three (the average number) recover, the recoveries were boasted of, as cures."

For a long time the small, the medium and the heroic dose doctors published how successful had been their practice. In time, however, this abuse of calomel declined, but it is not yet extinct among that numerous class of practitioners who cannot rise above the grade of routinists or mere medicators.

Again, Drs. E. and A. B. Whitney, in their treatise on the subject, page 112, speak as follows: "Has calomel any influence or power to arrest this disease, to quiet the nervous system, relieve the cramps or restore warmth to the body? Its specific action, as far as known, can have no tendency whatever to relieve the system in any essential particular, or stay the progress of disease, or delay its inevitable result, if it remains unsubdued by the action of other remedies.

Here we may ask, will opium aid or give the relief so urgently demanded? However serviceable as an astringent and anodyne in the premonitory stage of the disease, it cannot be exhibited in the second stage to so good an advantage, as, its direct influence is, to aid and promote congestions in those cases, where a tendency of this kind is already in existence.

Again, Dr. Hartshorn on cholera, page 65, says: "Unhesitatingly, I should hold the opinion that calomel is of no earthly use in cholera. The clinical experience, quoted for its success, is accounted for by the addition to it, almost always of opium in the prescription. Nor is the amount of success, with it, even then, great.

You observe that I have quoted three medical writers of quite recent times, who indeed are entirely averse to the treatment of cholera by calomel, with or without the addition of opium, for given reasons. Before I shall, however, proceed to say something as respects observations and facts relative to the employment of just these agents, I desire to lay before you the most positive convictions of no less high authorities, such as I consider rather advocates of this treatment.

Thus Prof. A. B. Palmer, who, according to his statements, had during three seasons, about two thousand cases under carefu observation and management in Chicago, in his remarks on Epidemic Cholera, page 19, says: "I am by no means insensible to the injurious effects, both proximate and remote, which the free use of mercury, under many circumstances produces. I have no sympathy with that class of practitioners, for ever seeing some 'liver complaint or bilious obstruction,' and hurling heroic doses of calomel or everlasting blue pills at these, so often imaginary difficulties. But mercury is a medicine of power, and has its uses, and cholera is one of the diseases where its remedial virtues are greatest. In this disease it seldom produces salivation, or other remote injurious consequences, and even if it did much more frequently, considering the extreme danger of the patient and the good effect it produces, we should be justified in its use. The use of this article (calomel) I consider of exceeding importance. My observations upon it have been careful and abundant, and I think I cannot be mistaken. The discharges may often be checked without its use, but unless the secretion of the liver is excited,

(and calomel when properly given and retained, tends powerfully and far more than any other article to excite that secretion,) the cholera discharges will again return and severe consequences follow. When mercurials, however, are used with opium, such returns of the symptoms are exceedingly rare."

He also says, page 32: "Some of the most remarkable recoveries of this kind, viz: the fully collapsed state, which I have witnessed, have been after large and repeated doses of calomel."

Further: Dr. N. L. North, on Epidemic Cholera, p. 24, remarks: "The calomel treatment, according to the reports of those who have used it, whether in larger or smaller doses, has produced better results than almost any other treatment, and it is said the patients are not apt to have consecutive fever, and that they are generally well in from three to five days."

Prof. Thos. F. Rochester, of this city, in a very ingenious and well-digested article, entitled "A few remarks on Cholera, (*Buffalo Medical & Surgical Journal*, vol. vi, Aug. 1866,) in respect to the treatment of calomel, page 4, expresses himself thus: "If the writer indicates a plan of treatment to be generally followed, he does so in no spirit of egotism, and with all respect to the opinion of those entertaining different views. It is the result of extended personal observation and experience; he has tried and has seen tried very many methods of treatment, and while he candidly admits that nothing is very reliable or satisfactory, his decided preference is as follows," etc.

And further, he adds: "It has been argued that calomel should not be given in the manner advocated on account of the great liability of salivation. It will salivate but very few, but if it is potent in permanently arresting the serous diarrhœa, and also in arresting some of the choleraic sequelæ, salivation is of minor importance."

What, however, has by distinguished modern observers been said, either to favor or condemn the use of calomel in this disease, the same applies also to the employment of opium.

Prof. Maclean, cited by Drs. E. and A. B. Whitney, in their treatise on Asiatic Cholera, page 66, remarks: "Opium in cholera should be given only in the premonitory diarrhœa. At this stage in combination with a stimulant, it is of the highest value. If persevered in, particularly in the strong doses, it is a dangerous remedy, inducing fatal narcotism, at least, interfering with the functions of the kidneys, and so leading directly to uræmic poisoning."

Dr. Nelson, whilst he, as we have seen, in the strongest terms condemns the use of calomel in cholera, seems to be an enthusiast, however, as regards opium. In his monograph, page 188, he says: "Throughout the whole reign of cholera, opium has been had recourse to, with benefit, when judiciously employed, but which, alas, has seldom been the case, for there are only a few practitioners who are deeply versed in the recondite action of this heroic remedy, while the great majority see in it only an every day drug, and are totally ignorant of its mysterious power; a larger dose than one grain might so paralyze the stomach as to arrest its power of ejecting what ought to be discharged and lock it in, to the injury of the patient"

Again, page 190, he observes: "When opium is administered at the proper time and in proper quantities, it will not only allay a state of vomiting, which is not longer needed, but it will also sooth the whole economy in a notable degree and pave the way to a recovery."

Dr. Nelson here doubtless means the use of opium in small or medium doses in the second stage of cholera.

Drs. A. and E. B. Whitney, although according to page 112 of their treatise, they really appear as sharply opposed to the employment of opium in the second stage of the disease, as you will please to recollect, nevertheless they at p. 191, strangely enough, advise the temporary addition of tinct. opii to certain injections into the bowels in cases in which on account of the great irritability of the stomach, their favorite combination of chloroform cannot be retained.

Prof. Palmer, in his remarks on Epidemic Cholera, p. 17, says: "Opium in proper doses and combination in certain stages of cholera, before the vital powers are much exhausted, and while irritation of the stomach and bowels is the most prominent symptom, is the great remedy in the disease, or at least one of the prominent and essential items of a correct treatment. It is by far the most potent remedy we possess for allaying that irritation, arresting the flow of fluids to the mucous surface and controlling the debilitating discharges, and when from its use these effects are produced, the system by other proper aids, is generally enabled to rally and struggle successfully against the morbid influences." He also says: "No language can be too strong in condemning the use in larger quantities in the advanced stages of the disease."

Dr. Blacklock regards the exhibition of opium as a poison in cholera.

Dr. Cox, quoted by Dr. Peters in his treatise, p. 153, remarks: "It is quite powerless to check the vomiting or purging, or to relieve the cramps, and is totally inadmissible in any stage or any dose."

Dr. Warring says: "Dr. Cox's opinion is perhaps too sweeping, but that it is injurious in large and frequent doses, either alone, or in combination, is a fact that few will be inclined to doubt."

I could quote a great many more medical writers, either advocates or opponents to the employment of opium alone or combined; for my present purpose, however, the number cited above seems to be quite sufficient. One could believe that physicians might be rendered skeptical in medical science by such conflicting statements and opinions, but it strikes me the profession will, on the contrary, but improve in this respect. We learn thereby a great truth, that opium in cholera has been abused, that it had been given in too large doses, or when in small quantities, its use too long persevered in and administered even frequently in impending and complete collapse; in short, that it had often been given without a clear view of the indications which this medicine fulfills. Exhibited in such improper doses and at improper times, opium has doubtless proved to be a very dangerous agent, and must have fearfully added to the bill of mortality in most of the irruptions of a disease which of itself appears so appalling and destructive in its effects. On the other hand we find the great practical utility of this remedy well established and recommended in cholera by such medical men, who have always made a wise and careful use of it in proper quantities and at proper stages of disease, closely watching its effects on the system.

We finally observe with some surprise and satisfaction, that even such authors as in principle as well as practice, are opposed to its employment in cholera, under certain circumstances, however, seem to be forced to its use and advise it in such cases in spite of all their *a priori* reasoning.

Mr. President and gentlemen of the Association:—After having tried various plans of treatment during the last three epidemics of cholera in our city, I feel it my duty to state, that none has given me such entire satisfaction as what has been termed the treatment of cholera by calomel and opium conjointly with stimulants; and this evening I feel indeed happy when I am enabled to recount to you the history of Mr. P. B., whose life had three times been in extreme danger in the cholera epidemic of 1854, but who fortunately escaped each time by being subjected to this mode of treatment. These three severe attacks succeeded one another with fourteen days' interval of apparently restored health. The two latter attacks taking place soon after great imprudence in diet, as I learned from his wife. In the attacks in which I have seen him, strange as it may appear, always in a more or less collapsed state, his pulse and heart sounds were then almost imperceptible, respiration greatly oppressed, his voice in the second and third attack nearly extinct, *vox cholerica*, his tongue and general surface icy cold, the latter in the third attack covered with clammy perspiration, shriveled and corrugated, and at the extremities already cyanosed. His features had become ghastly. He likewise presented in each of these states, alternately, a picture of great restlessness, or of entire apathy and indifference, only craving for drinks; in fact, a series of phenomena were exhibited which could only be the result of the powerful action of the cholera poison, due to the great loss of fluids from the system by the incessant and profuse vomiting and purging and the severe cramps he had to endure. This patient afterwards enjoyed a full, vigorous health up to this present day. Indeed he represents a true type of the Teutonic race. I could also report the cases of two married ladies who were twice and other patients who were once in such a dangerous condition during the two latter epidemics in this city, having recovered under the adopted plan of treatment.

I shall proceed now to describe in detail the method I have pursued, and which in good constitutions afforded encouraging results. For instance, I visited an adult person in the second stage, greatly suffering from copious vomiting and so-called rice-water discharges, cramps of the extremities, etc., I gave my patient a powder composed of calomel 20 grains, opium 1 grain, ipecac ½ grain, in a teaspoonful of sweetened water, a small piece of ice being added. Did the stomach eject the medicine immediately, or a few moments afterwards, the same dose was re-administered. Ice was ordered to be taken into the mouth *ad libitum*, in preference to drinking much water, which usually was thrown off as soon as it reached the stomach. At reasonable intervals a teaspoonful of brandy also, with the addition of ice was given to the patient, until contra-indicated. After the lapse of two hours a second powder was exhibited.

I shall state as a fact that in the majority of cases in which the first dose of the comp. calomel powder had been retained for about fifteen minutes, the vomiting, as a rule, ceased, the profuse rice-water evacuations gradually diminished in copiousness and frequency, until after the lapse of several hours a less exhausting bile-colored diarrhœa became established, which finally disappeared under the further use of a few fractional doses of powdered opium and ipecac, $\frac{1}{8}$ of a grain of each. In exceptional cases of a very grave character, more than two, compound calomel powders were found necessary, the addition of opium to calomel of the composition mentioned, must be called a mere medium dose. In such a combination opium, to which ipecac is added, will produce no deleterious effect on the brain, while it will suffice to act satisfactorily as an anodyne and sedative, to allay the great irritation of the stomach and intestinal tube.

I should here mention that in a number of instances the gums of the patient after the expiration of ten to twelve or more hours, appeared to be more or less swollen and somewhat painful; in some cases salivation was produced, which, however, yielded in the course of a week to very small doses of iodine combined with iodide of potassa, and a gargle containing a small amount of diluted hydro chloric acid. Warm stimulating poultices were placed around the limbs suffering from cramps, and also over the bowels, which seemed to add at least to the relief of the pain more than sinapisms or repeated rough frictions made by the bystanders, which under the circumstances even did harm. As soon as the vomiting ceased the patients were supported by proper nutriment in small quantities, spoonful by spoonful, at reasonable intervals, as chicken-broth, beef-tea, or also milk with a little soda water, etc. This principally constituted the medication in the second stage of cholera. If the patient was seen in the condition of impending collapse, and the dejections were still continuing, the same treatment was pursued, together with the occasional employment of spirits of camphor, tinct. of capsium, etc., and also aided by the application of artificial warmth to the surface until re-action became established, if possible. In complete collapse the patient was rather left to the efforts of nature, than of being subjected to active treatment. Good results have followed some times by the continued use of small pieces of ice and beef-tea.

What by the lamented Dr. Newman in his able paper (published 1854) had been termed "congestion of the brain," and spoken of as a stage of cholera, dependent upon irritation of this organ, excited in the course of the disease, and which to others and my own experience, will be observed in one epidemic more than another, or also may fail to make its appearance, has been treated by me by ordering cold application to the scalp, one wet cup behind each ear, (in two instances,) one grain of calomel to be given every two hours, and as much ice placed on the tongue as the patient desired, until all dangerous and distressing symptoms seemed to subside.

I must, however, mention that of the cases which came under my observation, (five in number,) three had been under the care of Eclectics, having been treated chiefly with tablespoon doses of powdered ginger, but which were always ejected from the stomach; while the remaining cases had received no medical attendance previous to my being called. All these patients, among whom was a woman who shortly before had given birth to twins, and having had no medical aid during her suffering from cholera, soon did well. In the treatment of these cases as an average, six grains of calomel together with the applications mentioned, was usually sufficient to relieve the congested state of the brain.

After having described the mode of treatment I have pursued, especially during the two latter cholera epidemics, I shall now attempt to point out the probable action of the remedies under consideration. Calomel combined with opium in proportions already mentioned, appears to act first as a sedative, and second to produce a certain alterative effect on the system in general, but not as has been assumed, to act as a chologogue, a mere restorer of biliary secretion, since the gall-bladder has been found filled with bile after death. The combination of calomel and opium seems rather to restrain and effectually check not only vomiting, especially with the frequent administration of small quantities of ice, but also to arrest more or less rapidly the profuse, so-called rice-water dejections, and finally change them into healthy stools. Even when a certain collapsed state has taken place, re-action has by the judicious use of calomel sometimes been established, however hopeless the condition of the patient did appear. In such cases animal heat will gradually re-appear, the general surface lose its death-like feeling, the circulation be restored and become more and more equalized; a warm moisture will take the place of the cold condition of the skin, and with the resumption of the pulmonary function of æration, the restlessness will vanish, and the voice, before husky or nearly extinct, gradually becomes natural, the sensation of oppression of the precordia, the insatiable thirst, the sharp and contracted state of the features will disappear; also the lost nerve power, the secretions, especially that of urine be restored, etc.

Finally, since it cannot be denied that the deleterious effects of the cholera poison are decidedly manifested upon the great sympathetic and pneumo-gastric nerves, in consequence of which their functions appear to be seriously disturbed or even suspended, and since also a great many good results have in epidemic cholera been observed from the employment of calomel and opium conjointly, I may perhaps be permitted to conclude that it possibly may exert a certain beneficial, although up to the present time but little understood influence over the organic nervous system, probably sedative and alterative in its effects, in consequence of which a salutary change of the disturbed functions of organic life and even of the deranged chemico-vital condition of the blood itself might be established, and thus pave the way to a final restoration of health. This must, however, be regarded as a mere hypothesis; indeed various articles of the materia medica employed in cholera, in very different ways and modes, have undoubtedly produced very happy results.

Mr. President and gentlemen of the Association:—When during the last three or four years I have read articles or treatises on epidemic cholera, I confess it has often been with mixed feelings of joyful surprise and also of sorrow, for very obvious reasons.

While we have not yet found the means of preventing entirely the spread and dreadful ravages of this deadly pestilence, yet more has been done in this direction by the medical profession during the just mentioned period than ever before.

As to the treatment of cholera, it must be admitted no remedy whatever seems to have fully satisfied the medical mind up to this very day. Of late several agents have strongly been recommended by some observers, especially for the purpose of a more speedy relief of the severe spasms and cramps in the second stage, as the employment of hypodermic injections of morphia, with or without the addition of atropia, or the internal use of chloroform; the latter in certain combinations is also said by some to rank first among means to meet powerfully and fulfill most promptly the indications of any stage, and even that of the "algide condition," and to succeed in effecting a cure in nine-tenths of cases of those attacked.

Still I should mention that the same success in treating Asiatic cholera is claimed by Prof. Palmer of Chicago, an eloquent advocate of the calomel treatment, according to his own plan. It may however be truly said, that with but few exceptions, nearly every writer on the topic which is still involved in so much obscurity, prefers to recommend his own favorite mode of treatment, and takes pains to criticise all, or most others, or even ventures to condemn them more or less, not only as irrational and unsound in principle, but also as quite ineffectual in practice, which expressions cannot in fact always receive the sanction of a profession so enlightened and high-minded as the medical. Indeed, we as the friends of suffering humanity, must content ourselves with the consciousness of having done our full share of duty in having attempted to save lives in the darkest hours of peril, always to the best of our ability and conviction, in spite of the great discrepancy of opinions and theories. And should it seem to be proper to defend our modes of treatment, we should not hesitate, we must do so in honor to the profession at large and medical science.

The President, Dr. Eastman, having arrived, took the chair.

Dr. Miner moved a vote of thanks to Dr. H. Nichell for his well-considered and able essay.

Dr. Eastman suggested that the motion include the request of a copy of the essay for publication.

Dr. Miner replied, that he preferred not to put the motion in that form; not however because he did not think the author deserving the compliment, but because it is entirely unnecessary, for the reason that all papers read before the Association, and worthy of publication, will be gladly published as proceedings of the Association without a special request. Voting to have them published could only add to the embarrassment of the editor of the Journal, who must exercise some discrimination in selecting the material composing the pages of the Journal. Papers and communications of interest locally, and not very objectionable when presented before the members of the Society only, were sometimes of no general interest to the profession, and when published, were not only of no value, but exposed the Society and author to derision and ridicule. Nothing was more unpleasant, than to reject such papers when designed for publication.

Dr. Eastman replied at some length, sustaining Dr. Miner in his views, and justifying and approving his position, regarding it as not only proper but absolutely indispensable both for the credit of the Journal and the respectability of the Society that most careful supervision be exercised in this respect. The Secretary reports the proceedings, and cannot be expected to reject or change written papers presented by members. The editor of the Journal must exercise this power, and should be sustained and aided in an effort so manifestly important, not only to the Journal, but to the credit of the Association and reputation of its members.

The motion of Dr. Miner was carried.

Scarlatina and diphtheria were reported as prevailing.

Adjourned. T. M. Johnson, Sec'y.

---

*Position in the Reduction of Inguinal Hernia.*—Dr. Bond, of Nova Scotia, writes to the London Medical Times and Gazette, that he has never failed in the reduction of inguinal hernia when the patient stood erect, even after previous long continued, but unsuccessful attempts in a recumbent position. His attention was first called to this position by having a patient who was unable to reduce his own hernia except when standing. Dr. Bond offers no theory on the subject, but states that it is not on account of the erect position causing syncope.

